# Anti-apoptotic genes are synergistically activated in OVSAYO cells cultured under conditions of serum starvation and hypoxia

**DOI:** 10.1016/j.gdata.2015.05.029

**Published:** 2015-06-03

**Authors:** Shiro Koizume, Yohei Miyagi

**Affiliations:** Molecular Pathology & Genetics Division, Kanagawa Cancer Center Research Institute, 2-3-2 Nakao, Asahi-ku, Yokohama 241-8515, Japan

**Keywords:** Hypoxia, Ovarian cancer, Sp1, ICAM-1, Anti-apoptotic genes

## Abstract

The tumor microenvironment is generally hypoxic because of the limited oxygen supply from inefficient or insufficient vasculature. Hypoxic tumor tissues are also poorly supplied with serum components. We have previously demonstrated that expression of the *FVII* gene is induced in response to hypoxia in ovarian clear cell carcinoma (CCC) cells. This gene activation is synergistically enhanced when cells are simultaneously subjected to serum starvation, and is dependent on the transcription factor Sp1 directly associating with the *FVII* promoter. We have identified additional genes activated via a similar Sp1-dependent mechanism by conducting cDNA microarray analysis (GSE55565). *ICAM1*, which encodes intercellular adhesion molecule-1 (ICAM-1), is one such gene. ICAM-1 confers an anti-apoptotic effect upon CCC cells in vitro and promotes growth of CCC tumors. Here we describe the transcriptome analysis performed in our recently published study (Koizume et al., 2015). We further show that autonomous activation of the TNFα–NFκB axis is responsible for the synergistic activation of *ICAM1* under hypoxic and serum starvation conditions. This study provides additional information as to how CCC cell survival can be facilitated under conditions of serum starvation and hypoxia.

**Table:** 

Specifications	
Organism/cell line/tissue	OVSAYO human ovarian clear cell carcinoma cell line
Sex	Female
Sequencer or array type	Agilent 018450 Whole Human Gene Genome microarray, NimbleGen 2006-08-03_HG18_60mer_expr array
Data format	Raw and analyzed
Experimental factors	OVSAYO cells exposed to hypoxia or CoCl_2_
Experimental features	Total RNA isolated from OVSAYO cells cultured under hypoxic conditions or with CoCl_2_ stimulation was used for cDNA microarray analysis
Consent	N/A
Sample source location	N/A

## 1 Direct link to deposited data

http://www.ncbi.nlm.nih.gov/geo/query/acc.cgi?acc=GSE55565.

## 2 Experimental design, materials and methods

Cells of the ovarian clear-cell carcinoma (CCC) cell line OVSAYO were routinely cultured in RPMI 1640 medium supplemented with 10% fetal calf serum (FCS).

### 2.1 Identification of genes activated in an Sp1-dependent manner following CoCl_2_ treatment of OVSAYO cells

We identified genes in addition to *FVII* that are activated in an Sp1-dependent manner under hypoxic conditions [1]. We transfected CCC OVSAYO cells with non-specific (NS) or Sp1 targeted small interference RNAs (siRNAs) [2]. Culture medium was replaced 40 h post-transfection and cells were cultured for an additional 4 h in the presence of vehicle (water) or 500 μM CoCl_2_. Four experimental conditions were examined: NS-siRNA transfected with and without CoCl_2_, and Sp1-siRNA transfected with and without CoCl_2_ [2]. Total RNA was isolated using the SV total RNA isolation kit (Promega, Madison, WI, USA) according to the manufacturer's protocol. Microarray analysis including RNA quality assessment, labeling, hybridization, and data analysis was performed under contract service by Dragon Genomics Center (Takara Bio, Mie, Japan). Cy3-labeled cRNA was prepared using 500 ng RNA with the Quick Amp labeling Kit, one-color (Agilent, Santa Clara, CA, USA) according to the manufacturer's recommendation. Cy3-labeled cRNA was fragmented and hybridized to Agilent 018450 Whole Human Gene Genome microarray (4x44K v2, G4112F) at 65 °C for 17 h with rotation and then washed. Experiments were performed using the Gene Expression Hybridization Kit (Agilent) and Gene Expression Wash Pack (Agilent) according to the manufacturer's recommendation. Slides were scanned using an Agilent DNA Microarray Scanner and data were processed using Agilent Feature Extraction 7 software. Feature and background regions were determined using the CookieCutter segmentation algorithm to obtain processed signal intensities. Background was subtracted (Spatial Detrending) and then, signal intensity of total array was corrected (Multiplicative Detrending).

### 2.2 Identification of genes synergistically activated in OVSAYO cells under conditions of serum starvation and hypoxia

We identified genes activated in an Sp1-dependent manner in OVSAYO cells upon exposure to both serum starvation and hypoxia (1% O_2_; SSH) conditions using transcriptome analysis following cDNA microarray experiments. OVSAYO cells were cultured under routine conditions for 24 h and then exposed to one of four experimental conditions. Cells were cultured for a further 16 h under conditions of normoxia with and without 10% FCS, and hypoxia with and without 10% FCS. Total RNA was isolated as described above. Labeling with Cy3 was performed using the NimbleGen one-color DNA labeling kit (Roche, Indianapolis, IN, USA) according to a recommended protocol provided with the gene expression microarray. Hybridization to 2006-08-03_HG18_60mer_expr array (Euk expr 385K catalog Arr Del) was performed using the NimbleGen Hybridization Kit (Roche, Madison, WI, USA) according to the manufacturer's instructions. Scanning was conducted using a GenePix Personal 4100A instrument (Axon Instruments, Sunnyvale, CA, USA) and images were processed by NimbleScan v2.6 software (Roche) to obtain raw data (pair) files. Data were further processed using the same software to obtain normalized data. These data were analyzed using a contract service provided by Subio Inc. (Kagoshima, Japan, www.subio.jp/) using Subio Platform v 1.14 software.

### Identification of anti-apoptotic genes synergistically activated with ICAM-1 under SSH conditions in OVSAYO cells

**Fig. 1:**
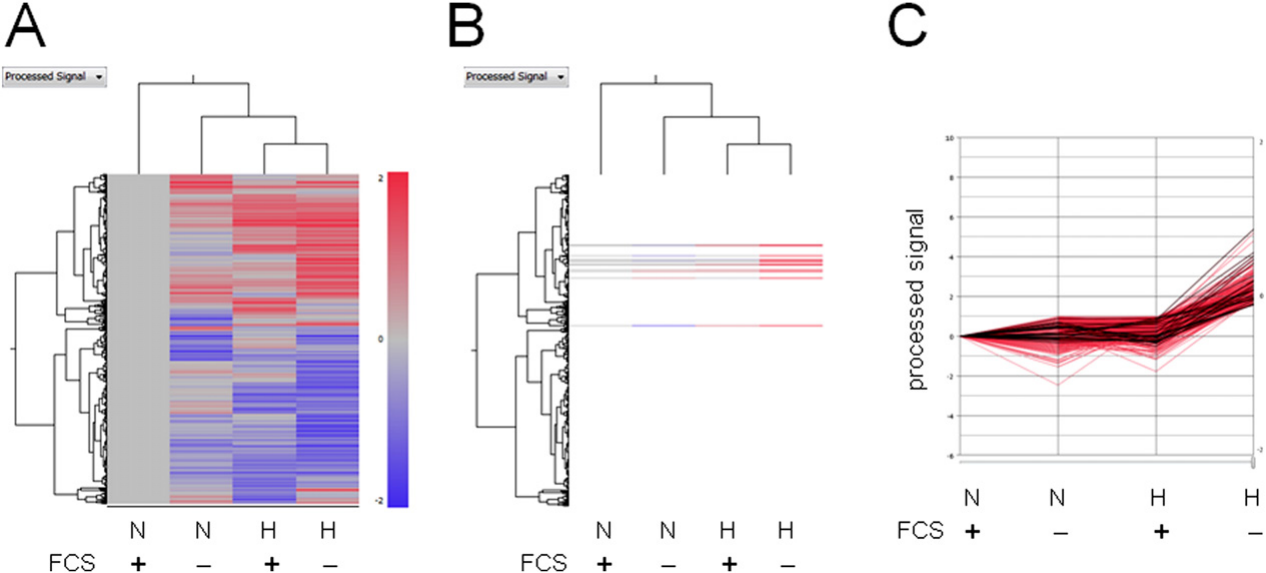
Identification of genes synergistically activated in OVSAYO cells cultured under SSH conditions for 16 h. A. Heat map representation of all measurements. B. Synergistically activated genes (401 total measurements) are highlighted. C. Line graph representation of the transcript levels of genes shown in B. Lines corresponding to expression of anti-apoptosis related genes are depicted in black. N and H are indicative of normoxia for 16 h and hypoxia (1% O_2_) for 16 h, respectively.

**Fig. 2:**
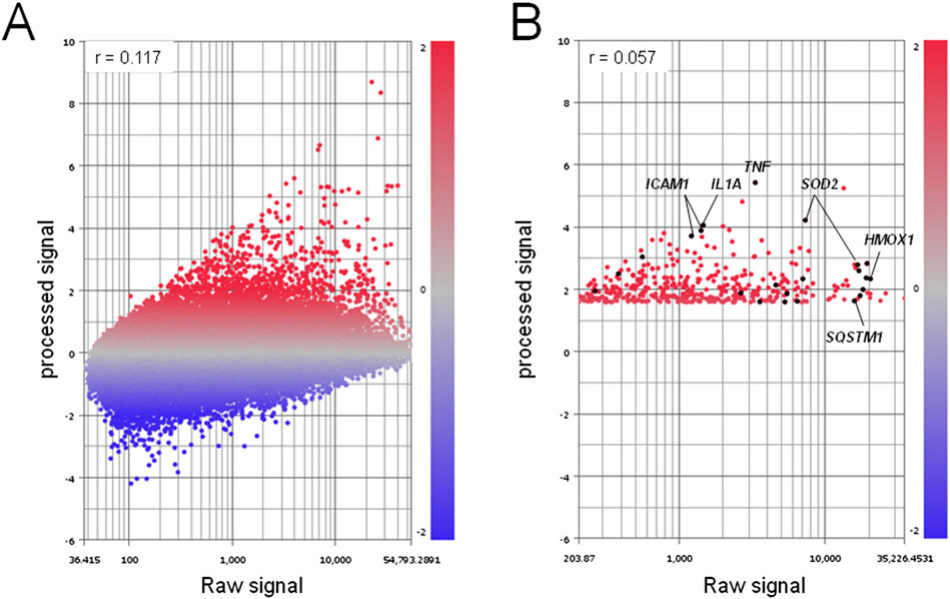
Scatter plot representation of gene expression levels (raw signal vs. processed signal) regulated in OVSAYO cells cultured under SSH condition for 16 h. A. All measurements. B. The 401 candidate probes shown in Fig. 1 were selected. Plots corresponding to expression of anti-apoptotic genes are highlighted in black. Genes of particular interest, including *ICAM1*, are indicated.

We searched for anti-apoptotic genes that were synergistically activated with *ICAM1* under SSH conditions using Subio platform software. The ratio of genes with increased expression under SSH compared with that of normoxia with FCS was > 3. Meanwhile, a ratio of < 2 resulted from comparison of gene induction under normoxia without FCS with hypoxia in the presence of FCS [2]. Venn diagram [2], heat map, and line graph representations (Fig. 1A–C) demonstrate the 401 (of 47,633 total) probes that were synergistically activated under SSH conditions. Scatter plot representations of the data sets shown in Fig. 1 reveal that raw signals do not significantly correlate with processed signals (Fig. 2A and B). Further extraction of genes revealed that 11 individual genes (21 total probes) were associated with anti-apoptotic activity (Table 1, designated with gray background). Further pathway analysis conducted using the Kyoto Encyclopedia of Genes and Genomes (KEGG) and DAVID bioinformatic database (http://david.abcc.ncifcrf.gov/home.jsp) revealed that the IL-1 and TNFα–NFκB pathways may be involved in cell survival. We then demonstrated that the TNFα–NFκB axis was indeed responsible for the synergistic activation of *ICAM1* in OVSAYO cells [2].

Additional analysis of genes with increased expression under SSH conditions compared with either normoxia without FCS or hypoxia with FCS identified 688 probes. Identical analysis to that described above revealed 24 genes (41 measurements) which were potentially associated with anti-apoptotic effects (Table 1). KEGG pathway analysis identified additional anti-apoptotic factors including X-linked inhibitor of apoptosis and NFκB-inhibitor α as potentially involved in survival of OVSAYO cells exposed to SSH.

## 3 Discussion

We recently reported that transcription from *ICAM1* is robustly activated in CCC cells exposed to SSH conditions [2]. Intriguingly, unlike the conventional hypoxia-driven gene expression mechanism which is mediated by protein complex formation between hypoxia inducible factors (HIFs) and aryl hydrocarbon receptor nuclear translocator (ARNT), this transcriptional activation is independent of ARNT [2]. Rather, an interaction between HIF2α and Sp1 in association with the gene promoter region is essential for synergistic transcriptional activation [2]. Our study further demonstrated that an insufficient supply of long chain fatty acids (LCFA) can lead to synergistic gene activation [2]. Increased ICAM-1 protein levels confer a growth advantage upon CCC cells both in vitro and in vivo [2]. Notably, ICAM-1 also confers an anti-apoptotic effect on CCC cells as RNAi-mediated suppression of ICAM-1 promoted cleavage of poly(ADP-ribose) polymerase-1 and increased caspase activity in CCC cells cultured under SSH [2]. Presently, the mechanisms underlying the anti-apoptotic activity of ICAM-1 are unclear. However, soluble ICAM-1 can transmit signals on the surface of endothelial cells [3]. Moreover, ICAM-1 signaling by eosinophils during inflammation enhances survival through activation of the ERK signaling pathway [4]. Therefore, CCC cells may develop enhanced survival potential through ICAM1-driven activation of similar signaling cascades under SSH conditions. Our current findings provide an insight into how CCC cells could survive under severe hypoxic conditions with a limited supply of LCFA. Further study of the relationship between ICAM-1 induction and stimulation of anti-apoptosis signaling pathways will lead to greater understanding of the survival mechanisms employed by cancer cells in hypoxic tumor microenvironments.

## Conflict of interest

None declared.

## Figures and Tables

**Table 1:**
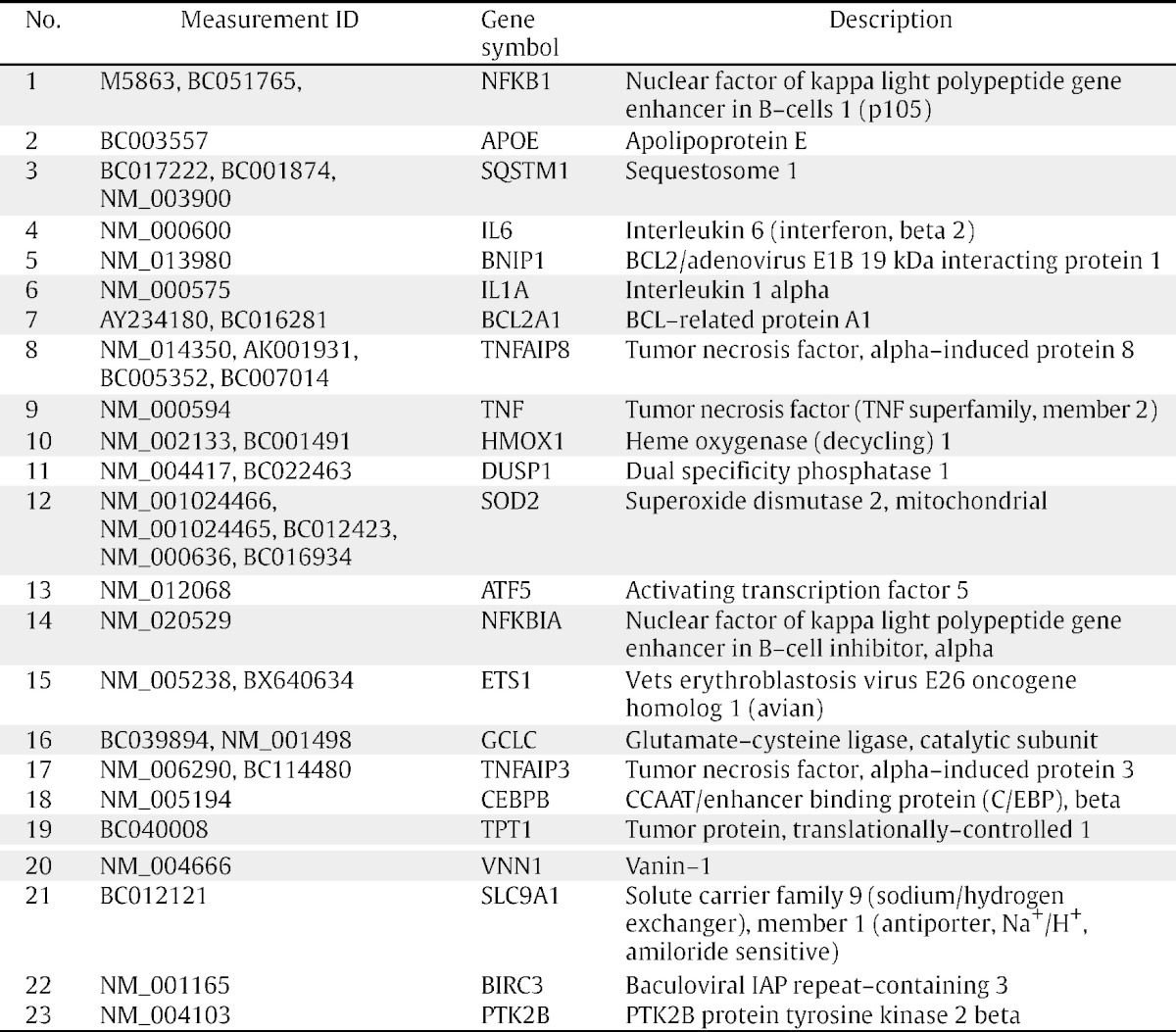
Anti-apoptotic genes synergistically activated in OVSAYO cells cultured under SSH conditions for 16 h. Genes depicted were selected from 688 measurements as described in the text. All anti-apoptotic genes extracted from 401 total measurements (Ref. 2 and Fig. 1B and C) are included in this table and are indicated with a gray background.
